# “Spidey Can”: Preliminary Evidence Showing Arachnophobia Symptom Reduction Due to Superhero Movie Exposure

**DOI:** 10.3389/fpsyt.2019.00354

**Published:** 2019-06-07

**Authors:** Yaakov S.G. Hoffman, Shani Pitcho-Prelorentzos, Lia Ring, Menachem Ben-Ezra

**Affiliations:** ^1^Interdisciplinary Department of Social Sciences, Bar-Ilan University, Ramat-Gan, Israel; ^2^School of Social Work, Ariel University, Ariel, Israel

**Keywords:** positive-exposure, Spider-Man, Ant-Man, comics, phobia, arachnophobia, movies, cognitive behavioral therapy

## Abstract

Fear of insects, mainly spiders, is considered one of the most common insect phobias. However, to date, no conducted studies have examined the effects of phobic stimulus exposure (spiders/ants) within the positive context of superhero movies, such as *Spider-Man* or *Ant-Man*. A convenience sample of 424 participants divided into four groups watched different clips. Two intervention groups (*Spider-Man*/*Ant-Man*) and two control groups (Marvel opening/natural scene) were measured twice (pre–post intervention). The measures comprised an online survey assessing socio-demographic variables, familiarity with superhero movies and comics, and phobic symptoms. Reduction in phobic symptoms was significant in the *Spider-Man* and *Ant-Man* groups in comparison to the control groups. Seven-second exposure to insect-specific stimuli within a positive context reduces the level of phobic symptoms. Incorporating exposure to short scenes from superhero movies within a therapeutic protocol for such phobias may have the potential to be robustly efficacious and enhance cooperation and motivation.

## Introduction

Since the beginning of the new millennium, along with the increased popularity of the internet and the rise of “geek culture” as part of a new technological era, superhero movies based on Marvel comics have become widely popular and have been integrated as part of mainstream culture. This phenomenon has undergone another boost, making it a worldwide phenomenon with the introduction of the Marvel Cinematic Universe (MCU). In these movies, insects (e.g., *Spider-Man*, *Ant-Man*) are shown in a positive wider context. The effect that such exposure may have on phobia symptoms, such as arachnophobia (phobia of spiders), has not been studied. Typically, cognitive behavioral therapy (CBT), where exposure is central, is the leading therapy for specific phobias, especially those related to insects (1). CBT may employ gradual exposure to phobic stimuli by introducing them in non-aversive and different contexts (2). Such exposure does not typically include a positive context. This raises the question of whether minimum exposure to phobic stimuli (i.e., spiders in the context of *Spider-Man* or ants in the context of *Ant-Man*) in a leisure, non-therapeutic environment will also reduce the level of phobic symptoms. Although movies are used in cinema therapy (3), the potential usage of positive exposure to phobic stimuli within the context of superhero movies has not been explored. Based on the underlying tenets of CBT exposure, we hypothesized that exposure to scenes depicting spiders/ants within a positive context (i.e., *Spider-Man*/*Ant-Man*) will robustly decrease the level of phobic symptoms, over and beyond the issue of whether participants are fans of superhero comics and movies who have viewed such movies and read the relevant comics. As this is a preliminary and novel study, we have outlined the design we used. There were four conditions: the first condition (group 1) comprised an intervention group where participants watched a clip from the Spider-Man movie. The second condition (group 2) also comprised an intervention, where participants viewed a short excerpt from the Ant-man movie. The third condition (group 3) was a control where participants watched a clip from Marvel’s classical movie opening. The fourth condition (group 4) was also a control where participants watched a clip of a natural scene of a meadow. All videos were 7 seconds long. Each group was measured twice, before and after watching the clips. Our hypothesis was based on the assumption that arachnophobia is one of the most common specific phobias (4); accordingly, the potential symptom reduction in this group should be the greatest, i.e., viewing a clip from *Spider-Man* should yield the highest reduction in phobic symptoms.

## Methods

### Participants and Procedure

An online quasi-intervention (before–after) study was conducted on 424 participants, who were recruited in a convenience sample *via* social media. The inclusion criteria were age over 18 and being proficient in Hebrew. The mean age was 32.07 (S.D. = 10.55, age range 18–71). The majority of the participants were women (n = 247, 58.3% of the sample). Each participant received one of four links, which were distributed *via* social media. Among the four study groups (two intervention groups and two control groups), groups 1 and 2 (intervention groups) were either exposed to spider scene from the Spider-Man 2002 movie (link: https://www.youtube.com/watch?v=pWPAnj6Si50) or to an ant’s scene from the Ant-Man 2015 movie (link: https://www.youtube.com/watch?v=GRuaBPgO8LY). Groups 3 and 4 were respectively exposed to either the Marvel classical movie opening (link: https://www.youtube.com/watch?v=1YXAsO0Tcls) or to a natural scene of a meadow (link: https://www.youtube.com/watch?v=1VUFQVan-Ww, original video: https://www.youtube.com/watch?v=MJ9-HmGWw1k). Each scene was seven seconds long. All groups answered the same survey twice. The study was approved by the last author’s university’s ethics committee.

### Measures

*Familiarity with superhero movies* was measured by a single item: “How familiar are you with Marvel movies?” This item was rated on a five-point Likert scale ranging from 1 (not at all) to 5 (very much).

*Familiarity with superhero comic literature* was measured by a single item: “How familiar are you with Marvel comics literature?” This item was rated on a five-point Likert scale ranging from 1 (not at all) to 5 (very much).

The familiarity questions were measured only at baseline.

*Phobic symptoms* were measured by the modified Severity Measure for Specific Phobia–Adult version (5) adjusted to the International Statistical Classification of Diseases and Related Health Problems (ICD) version 11 addressing insect phobia: For group 1, the questions were framed by instructing participants to address spiders (arachnophobia). In group 2, participants were instructed to rate the very same questions vis-à-vis fear of ants (myrmecophobia). For the remaining two control groups (groups 3 and 4), participants were asked to respond to these same phobia questions with regard to fear of insects (entomophobia). The selected four questions represent the core ICD-11 specific phobia items: “1. felt moments of sudden terror, fear, or fright; 2. felt anxious, worried, or nervous; 3. felt a racing heart, sweaty, trouble breathing, faint, or shaky; 4. avoided, or did not approach or enter, situations about which I worry”. Each item was rated on a five-point Likert scale ranging from 0 (never) to 4 (all the time). The total score for each participant ranged from 0 to 16. Phobic symptoms were measured at baseline and after watching the respective video clip. Cronbach’s alpha at baseline was 0.87; after watching the video clips, 0.89. In addition, we also created a phobia delta score composed of the baseline score minus the score after watching the respective video clip.

### Data Analysis

Before conducting data analysis, we removed participants below the age of 18 and those whose delta score deviated above or below four standard deviations. From the original sample of 451 participants, we excluded 27 participants. Simple paired t-tests were conducted in order to discern if there were differences within each group. Following the preliminary testing, the analytic plan comprised three main analyses (6). I) A one-way ANOVA analysis on the delta pre–post intervention levels of phobic symptoms across the four groups. II) A mixed-design repeated-measures ANOVA where both between-group and within-group effects were addressed. The independent variable was the group, coded as: 1, *Spider-Man* clip; 2, *Ant-Man* clip; 3, Marvel classic opening; and 4, meadow natural scene. The reason conditions 3 and 4 were included was to decouple the potential aspects of fun associated with a superhero movie or the relaxation element associated with watching any movie (a meadow) from exposure effects. The dependent variables were phobic symptoms at baseline and after watching the respective video clips. Age, gender, and familiarity with the *Spider-Man* and *Ant-Man* movies and comics were covariates. III) An analysis of covariance (ANCOVA) where the pre-intervention phobic symptom levels were covaried out, so that the sole dependent variable was the post-intervention phobic symptom levels. In addition, we obtained partial η^2^ and η^2^ as indicators of effect size (7), along with Cohen’s d (8). The data were analyzed by IBM SPSS version 25.

## Results

Starting with demographics, age differed significantly across groups. The mean age differed across the four groups, as revealed by a one-way ANOVA analysis [F(3,423) = 6.838; p < 0.001]. In intervention group 1 (*Spider-Man*) the mean age was 31.65 (SD = 9.91, in intervention group 2 (*Ant-Man*) the mean age was 29.57 (SD = 8.82), in control group 3 (Marvel classic movie opening) the mean age was 35.89 (SD = 11.36), and finally in control group 4 (neutral scene), the mean age was 31.40 (SD = 11.53).

The percentage of women did not differ among the groups. In intervention group 1 (*Spider-Man*), the percentage of women was 57.8%; in intervention group 2 (*Ant-Man*), the percentage of women was 63.9%; in control group 3 (Marvel classic movie opening), the percentage of women was 55%; and in control group 4 (neutral scene), the percentage of women was 55.5%. These differences were not significant using the Jonckheere–Terpstra test (std. J-T statistic = −.571; *p* = 0.568).

Familiarity with superhero movies did not differ significantly across groups. In intervention group 1 (*Spider-Man*), the mean score was 2.87 (SD = 1.35); in intervention group 2 (*Ant-Man*), the mean score was 3.03 (SD = 1.48); in control group 3 (Marvel classic movie opening), the mean score was 3.27 (SD = 1.39); and in control group 4 (neutral scene), the mean score was 3.17 (SD = 1.40). A one-way ANOVA revealed that these differences were not significant [F(3,423) = 1.729; *p* = 0.160].

Familiarity with superhero comics did not differ significantly across groups. In intervention group 1 (*Spider-Man*), the mean score was 2.13 (SD = 1.18); in intervention group 2 (*Ant-Man*), the mean score was 1.98 (SD = 1.07); in control group 3 (Marvel classic movie opening), the mean score was 2.17 (SD = 1.08); and in control group 4 (neutral scene), the mean score was 2.15 (SD = 1.13). A one-way ANOVA revealed that these differences were not significant [F(3,423) = 0.618; *p* = 0.604].

### T-Tests and One-Way ANOVA on the Delta Pre–Post Intervention Scores

Following the above, t-tests were separately conducted for each group in order to assess if there were any pre–post intervention differences. In intervention group 1 (*Spider-Man*), the mean phobic symptom level before and after exposure, was respectively 4.00 (SD = 4.12) and 2.84 (SD = 3.65). This reduction in phobic symptoms was significant (*t* = 4.843; *p* < 0.001). In intervention group 2 (*Ant-Man*), the mean phobic symptom level before exposure was 2.64 (SD = 3.53), after exposure it was 2.06 (SD = 3.39). This reduction in phobic symptoms was also significant (*t* = 2.781; p = 0.006). In control group 3 (Marvel classic movie opening), the mean phobic symptom level before exposure was 4.37 (SD = 4.00) whilst the mean phobic symptom level after the intervention was 4.06 (SD = 4.33). This slight reduction in phobic symptoms was not significant (*t* = 1.495; p = 0.138). Likewise, in control group 4 (natural scene), the mean phobic symptom level before (3.77, SD = 3.68) and after exposure (3.49, SD = 3.52) was similar and statistically different (*t* = 1.131; *p* = 0.262).

**Figure 1:**
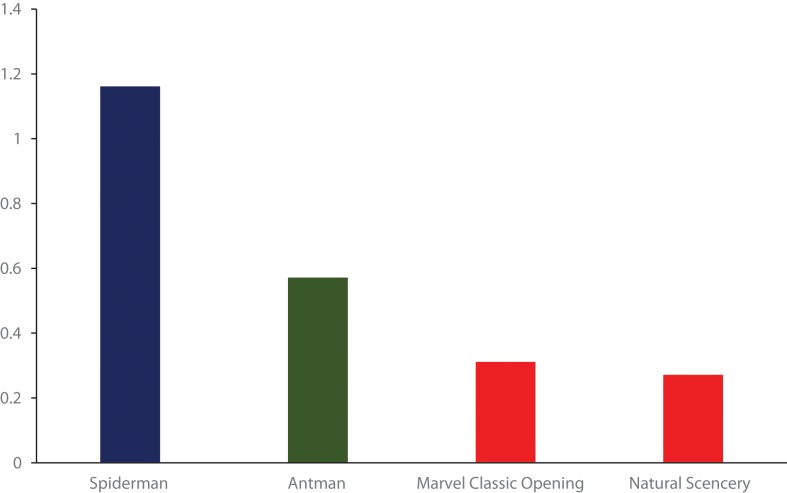
Reduction in phobic symptoms following the exposure to superhero movie scenes. (Bars represent delta between before and after symptom levels). ** The red bars are not significant; the green bar is significant (p = .006), and the blue bar is significant (p < .001).

The delta phobic symptom was largest in intervention group 1 (*Spider-Man*), with a mean of 1.16 (SD = 2.77), and to a lesser extent, in intervention group 2 (*Ant-Man*), with a mean of 0.57 (SD = 2.15); it was even lower in control group 3 (Marvel classic movie opening), with a mean of 0.31 (SD 2.07), and in control group 4 (natural scene), the mean was 0.27 (SD = 2.16). These pre–post intervention delta scores were subjected to a one-way ANOVA and were found to be significant (F(3,423) = 3.51; p = 0.015). See **Figure 1**.

### Repeated-Measures ANOVA

A repeated-measures ANOVA revealed the critical Group (1–4) X Measure (pre-post) interaction effect on phobic symptom levels was significant (F = 3.573, p = 0.014, partial η^2^ = 0.025). Likewise, the group main-effect was also significant (F = 9.250, p < 0.001, partial η^2^ = 0.063). The before–after difference in the phobic symptom scores was significant in the intervention groups but not in the control groups. Thus, although there was a decline in the level of phobic symptoms across all groups (see **Table 1**), this reduction was significant only for the intervention groups exposed to *Spider-Man* and *Ant-Man*, but not in the control groups. We also obtained the same results when controlling for age, sex, and familiarity with superhero comic literature and movies. Finally, the interaction effect size was slightly larger than a small effect, with a η^2^ of 0.0242 and Cohen’s d = 0.315. The between-participant effect size for the group main-effect was larger and approached a medium effect size, η^2^ =0.054, Cohen’s d = 0.484.

**Table 1:** Phobic symptoms before and after exposure to movie scenes across groups.

	Group 1–Intervention *Arachnophobia* (scene taken from Spiderman movie)	Group 2–Intervention *Myrmecophobia* (scene taken from Antman movie)	Group 3–Control *Entomophobia* (taken from classic marvel opening movie)	Group 4–Control *Entomophobia* (taken from movie scene of a meadow)	Repeated-measures between-group effects
Group	(N = 135)	(N = 108)	(N = 100)	(N = 81)	F = 9.250***; partial η^2^ = .063
Age, years	31.65 (9.91)	29.57 (8.82)	35.89 (11.36)	31.40 (11.53)	F = 15.271***; partial η^2^ = .035
Sex, female	78 (57.8%)	69 (63.9%)	55 (55%)	45 (55.5%)	F = 35.651***; partial η^2^ = .079
Familiarity with superhero movies	2.87 (1.35)	3.03 (1.48)	3.27 (1.39)	3.17 (1.40)	F = .865; partial η^2^ = .002
Familiarity with superhero comics	2.13 (1.18)	1.98 (1.07)	2.17 (1.08)	2.15 (1.13)	F = .02; partial η^2^ = <.001
Phobic symptoms before exposure	4.00 (4.12)	2.64 (3.53)	4.37 (4.00)	3.77 (3.68)	
Phobic symptoms after exposure	2.84 (3.65)	2.06 (3.39)	4.06 (4.33)	3.49 (3.52)	
Paired t-test for each group	t = 4.843***	t = 2.781**	t = 1.495	t = 1.131	
Δ Phobic symptoms	1.16 (2.77)	.57 (2.15)	.31 (2.07)	.27 (2.16)	
Between-groups ANOVA		F = 3.512*			

*p < 0.05. **p < 0.01. ***p < 0.001.

### ANCOVA

In addition, we ran an ANCOVA analysis, where the pre-intervention phobia symptom levels prior to the video exposure were covaried out, in order to further flush out effects. The dependent variable was phobia symptom levels after video exposure. We also ran this analysis when age, sex, and familiarity with superhero comic literature and movies served as additional covariates; results were the same.

### Unadjusted and Adjusted Mean Differences for Each Group

The unadjusted mean difference in intervention group 1 (*Spider-Man*) was 1.16 (SD = 2.77) (n = 135), while the adjusted mean difference in this group was 1.18 (SE = 0.20) (95% C.I. = 0.780–1.571). The unadjusted mean difference in intervention group 2 (*Ant-Man*) was 0.57 (SD = 2.15) (n = 108), while the adjusted mean difference in this group was 0.45 (SE = 0.23) (95% C.I. = 0.004–0.891). The unadjusted mean difference in control group 3 (Marvel opening) was 0.31 (SD = 2.07) (n = 100), while the adjusted mean difference in this control group was 0.43 (SE = 0.24) (95% C.I. = −0.040–0.894). The unadjusted mean difference in control group 4 (natural scene) was 0.27 (SD = 2.16) (n = 81), while the adjusted mean difference for this group was 0.26 (SE = 0.26) (95% C.I. = −0.244–0.769). The partial η^2^ effect size was 0.025, the η^2^ was 0.0242, and Cohen’s d was 0.315.

The ANCOVA results showed a significant effect for post-intervention phobia levels, where the group variable (F(3,416) = 4.04, *p* = 0.008) was significant. Follow-up Helmert contrast analyses revealed that although the two intervention groups, namely, *Spider-Man* (group 1) and Ant-Man (group 2), did not differ from each other (contrast estimate = .341, 95% CI = −.229 to .190, *p* = .24), only the Spider-Man intervention group (and not the Ant-Man group) significantly differed from control groups; it differed from both control group 3 (Marvel opening), contrast estimate = .881, 95% CI.302 to 1.461, p = .003, and from control group 4 (natural scene), contrast estimate = .847, 95% CI.240 to 1.455, p = .006. Yet, the group 2 (Ant-Man) effect was not statistically different from effects obtained in groups 3 (Marvel opening) and 4 (natural scene), contrast estimate = −.524, 95% CI −1.71 to .023, p = 0.061. This latter finding indicates that the observed effect in group 2 was weaker than that observed in group 1. Group1 (*Spider-Man*) was also different from all three groups, contrast estimate = −.690, 95% CI -1.143 to −.237, *p* = .003.

## Discussion

The results suggest that exposure to insect-specific stimuli within the positive, fantasy context of *Spider-Man* and *Ant-Man* reduces the level of phobic symptoms, despite being an *in vitro* exposure for a very short time duration of 7 s. This finding is important as *in vitro* exposure is usually less potent than *in vivo* exposure (9), yet since *in vivo* exposure can be difficult for some clients (10), it is often not applicable. Other less threatening forms of exposure, albeit less accessible, have thus been developed, such as virtual reality, which has been successfully used (11). Accordingly, showing robust arachnophobia symptom reduction resulting from accessible, very short *in vitro* exposure to spiders in the context of *Spider-Man* cannot be understated.

These results extend previous results showing that exposure to novel animals in neutral or fearful contexts can lead to different levels of self-rated fear (12). The current study is also in line with results showing that that while exposure to sad movies may elicit negative feelings, the *specific context* of this sadness (e.g., sad–avoidance–disgust vs. sad–attachment–tenderness) is critical in determining both the affective outcome and the changes in physiological arousal (13). Accordingly, it may be speculated that the disgust element associated with spiders may be mitigated in the context of *Spider-Man*.

## Limitations

Beyond the merits of the study, we advise caution, as there are several limitations that need to be acknowledged: 1) The effect that was measured was brief, and thus, how long such effects may endure, i.e., the long-term efficacy of these phobic symptom reductions, remains unknown. 2) Although group allocation was randomized, there is always a potential selection bias when using social media as a means of recruiting participants. 3) The sample was a non-clinical adult sample, and thus, it remains unknown if such results would replicate to clinical samples and to children. 4) Finally and importantly, although the results of the *Spider-Man* and *Ant-Man* groups showed the same pattern, these preliminary results would benefit from further replication.

## Future Studies

Further studies should expand these results by using a longitudinal experimental design with follow-up, in order to establish the time frame of the obtained effect. Future work should also focus on such exposure effects within phobic samples, across different ages, and within different therapeutic settings.

## Conclusion

In summary, the contribution of exposure to a positive context may be threefold: First, it seems to have a very high benefit-to-cost ratio; seven seconds of exposure yields more than a 20% arachnophobia symptom reduction. Such potent effects are speculated to derive from a positive (non-threatening) exposure. Accordingly, the idea that the current results may be relevant to exposure therapy for spiders (14, 15) is not trivial. Second, the current results open a novel theoretical direction, concerning potential effects of positive exposure, albeit in a fantasy context. Yet as the original phobia may not necessarily be rational, its exposure *via* fantasy movies may be a promising direction for other phobias as well. Third, although integration of such exposure (via *Spider-Man* and *Ant-Man*) into therapeutic protocols warrants future testing and consideration, exposure to stimuli within the context of superhero movies has the potential to increase motivation and adherence to an exposure protocol, which may be important to many clients for whom *in vivo* exposure to spiders may be difficult. For example, undergoing treatment for arachnophobia by watching a superhero clip may be experienced as fun and positive both by the client and by his/her peers. It may also be beneficial for reducing the stigma against psychological treatments and people with mental disorders (16).

In sum, pending future studies, it may be speculated that integrating superhero movie exposure into standard exposure treatments may constitute a novel direction that can enhance outcomes.

## Copyrights

The copyright for the excerpt from *Spider-Man* (2002) is owned by Columbia Pictures and Marvel Enterprises. The copyright for the excerpt from *Ant-Man* (2015) is owned by Marvel Studios. The Marvel classic opening copyright is owned by Marvel Studios. The copyright for the excerpt from the meadow scene is owned by 1HarryH.

## Data Availability Statement

The data will be sent upon request from MB-E or YH.

## Ethics Statement

The study was approved by Ariel University’s ethics committee. All the participants signed an electronic informed consent prior to engaging in the study. All the authors declare that we complied with ethical standards as stated above.

All procedures performed in studies involving human participants were in accordance with the ethical standards of the institutional and/or national research committee and with the 1964 Helsinki Declaration and its later amendments or comparable ethical standards. The study was approved by Ariel University’s ethics committee. This article does not contain any studies with animals performed by any of the authors.

Informed consent was obtained from all individual participants included in the study.

## Author Contributions

MB-E and YH conceived the idea and developed the concept of the paper. MB-E, SP-P, and LR collected the data. MB-E and YH analyzed the data. MB-E and YH wrote the first draft. SP-P and LR critically reviewed and made significant contributions to the final version.

We declare having no conflict of interest or otherwise.

## Conflict of Interest Statement

The authors declare that the research was conducted in the absence of any commercial or financial relationships that could be construed as a potential conflict of interest.
